# Washed microbiota transplantation promotes homing of group 3 innate lymphoid cells to the liver via the CXCL16/CXCR6 axis: a potential treatment for metabolic-associated fatty liver disease

**DOI:** 10.1080/19490976.2024.2372881

**Published:** 2024-06-28

**Authors:** Hao-Jie Zhong, Yu-Pei Zhuang, Xinqiang Xie, Jia-Yin Song, Si-Qi Wang, Lei Wu, Yong-Qiang Zhan, Qingping Wu, Xing-Xiang He

**Affiliations:** a Department of Hepatobiliary and Pancreatic Surgery, The First Affiliated Hospital of Shenzhen University, Shenzhen Second People’s Hospital, Shenzhen, China; b Department of Gastroenterology, The First Affiliated Hospital of Guangdong Pharmaceutical University, Guangzhou, China; c Guangdong Provincial Key Laboratory of Microbial Safety and Health, State Key Laboratory of Applied Microbiology Southern China, Institute of Microbiology, Guangdong Academy of Sciences, Guangzhou, China; d Department of Oncology, Affiliated Hospital of Nanjing University of Chinese Medicine, Nanjing, China

**Keywords:** Chemokine receptor, fecal microbiota transplantation, group 3 innate lymphoid cells, gut microbiota, metabolic-associated fatty liver disease

## Abstract

Despite the observed decrease in liver fat associated with metabolic-associated fatty liver disease (MAFLD) in mice following fecal microbiota transplantation, the clinical effects and underlying mechanisms of washed microbiota transplantation (WMT), a refined method of fecal microbiota transplantation, for the treatment of MAFLD remain unclear. In this study, both patients and mice with MAFLD exhibit an altered gut microbiota composition. WMT increases the levels of beneficial bacteria, decreases the abundance of pathogenic bacteria, and reduces hepatic steatosis in MAFLD-affected patients and mice. Downregulation of the liver-homing chemokine receptor CXCR6 on ILC3s results in an atypical distribution of ILC3s in patients and mice with MAFLD, characterized by a significant reduction in ILC3s in the liver and an increase in ILC3s outside the liver. Moreover, disease severity is negatively correlated with the proportion of hepatic ILC3s. These hepatic ILC3s demonstrate a mitigating effect on hepatic steatosis through the release of IL-22. Mechanistically, WMT upregulates CXCR6 expression on ILC3s, thereby facilitating their migration to the liver of MAFLD mice *via* the CXCL16/CXCR6 axis, ultimately contributing to the amelioration of MAFLD. Overall, these findings highlight that WMT and targeting of liver-homing ILC3s could be promising strategies for the treatment of MAFLD.

## Introduction

Metabolic-associated fatty liver disease (MAFLD), a prevalent liver disease, affects approximately 39.22% of the global population.^1,2^ The condition encompasses various liver abnormalities, ranging from steatosis to steatohepatitis, cirrhosis, and hepatocellular carcinoma.^3^ Despite the association of MAFLD with increased risks of cardiovascular disease, malignancy, and overall mortality,^4^ no approved pharmacotherapy is currently available.^5^

The newly proposed concept of the gut-liver axis, which emphasizes the interaction between the gut microbiota and liver disease, has gained increasing attention.^6^ Studies on the gut microbiome of patients with MAFLD have revealed alterations in microbial composition,^7,8^ suggesting that manipulating the gut microbiome may be a promising therapeutic strategy for MAFLD. Despite reports of successful results in mouse models of MAFLD using microbiome-targeted therapies such as probiotics, prebiotics, and synbiotics,^9–11^ clinical trials in human patients have not shown any significant reduction in hepatic fat content.^12,13^ Considering the complexity of microbial-host interactions,^6^ a single microbiome-targeted intervention may be insufficient to reduce hepatic steatosis in patients with MAFLD.

Fecal microbiota transplantation (FMT) is a promising therapeutic approach for restoring the overall balance of intestinal microecology. This method involves the infusion of a fecal suspension from a healthy donor into the gastrointestinal tract of a patient for the treatment of dysbiosis-related diseases.^14,15^ Washed microbiota transplantation (WMT) is an improved version of FMT in which the gut microbiota is extracted using an automated purification system and washed repeatedly to reduce the risk of FMT-associated adverse effects (AEs).^16^ Although a significant decrease in liver fat has been observed in MAFLD mice received gut microbiota from healthy mice,^17^ the therapeutic effects and underlying mechanisms of WMT in patients with MAFLD remain unclear.

Group 3 innate lymphoid cells (ILC3s), a newly identified type of immune cells, are primarily located in the intestinal lamina propria and maintain close interactions with the gut microbiota.^18^ ILC3s have been implicated in the development of several liver diseases, including hepatic ischemia-reperfusion injury, liver fibrosis, and hepatocellular carcinoma.^19–21^ Moreover, a recent investigation revealed a significant reduction in the proportion of ILC3s within the ileocolonic mucosa of individuals with intestinal graft-versus-host disease. Notably, FMT treatment restores ILC3 levels.^22^ These findings imply that FMT may exert its therapeutic effects through regulation of ILC3s.

In the present study, CXCR6, as the highest expressed liver-homing chemokine receptor on the hepatic ILC3s in mice, was found to play a crucial role in regulating the migration of ILC3s to the liver. Specifically, downregulation of CXCR6 on hepatic ILC3s in MAFLD mice caused an atypical distribution of ILC3s, with a significant reduction in the liver and an increase outside the liver. WMT upregulated CXCR6 expression on ILC3s, facilitating their homing to the liver in MAFLD mice through the CXCL16/CXCR6 axis. Consequently, hepatic ILC3s alleviated hepatic steatosis and liver damage *via* the secretion of IL-22. These findings highlight the potential of WMT to attenuate MAFLD by promoting ILC3 migration into the liver. Therefore, the regulation of ILC3s homing to the liver shows promise as an effective therapeutic approach for patients with MAFLD.

## Results

### The composition and function of the gut microbiota are altered in both MAFLD patients and mice

**Figure 1.**
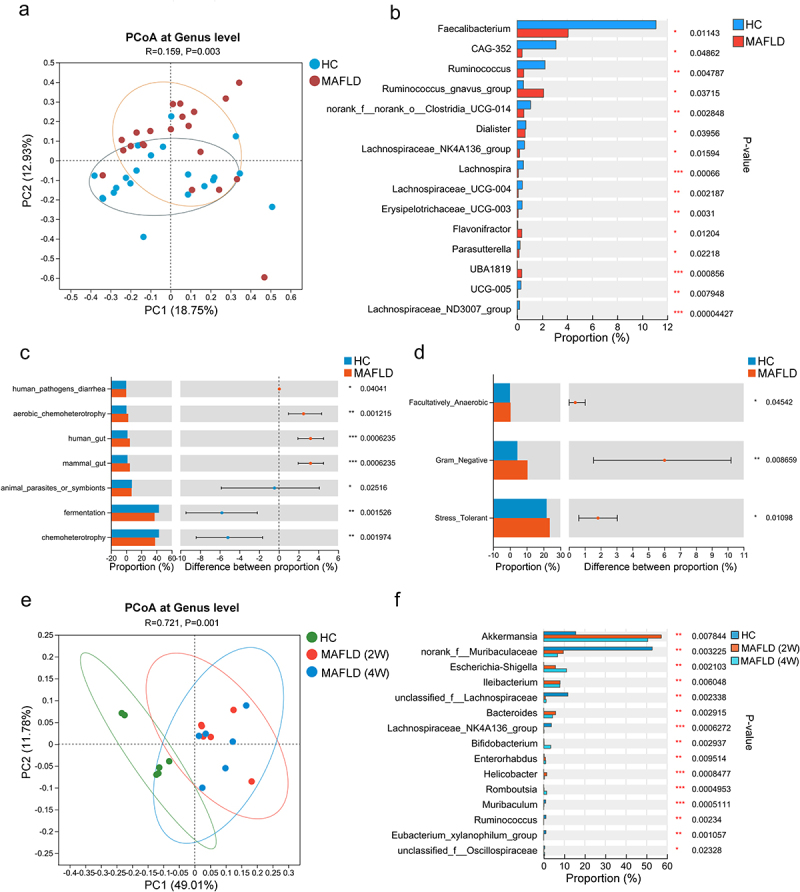
The composition and function of the gut microbiota were altered in both MAFLD patients and mice. (a) PCoA comparing microbial beta diversity between healthy controls (HC, *n* = 21) and patients with MAFLD (*n* = 21). (b) Bar plot showing the relative abundances of the top 15 differential genera in human patients. (c-d) Functional prediction analysis plots based on FAPROTAX and BugBase. (e) PCoA of microbial beta diversity in healthy (HC), MAFLD-2W, and MAFLD-4W mice. (f) Bar plot showing the relative abundances of the top 15 differential genera in mice. *n* = 6 mice per group. MAFLD, metabolic-associated fatty liver disease; PCoA, principal coordinate analysis. *p < 0.05; **p < 0.01; ***p < 0.001.

To compare the gut microbiota between patients with MAFLD and healthy controls, stool samples from 21 patients with MAFLD (age range 50.0 ± 13.0 years, 16 males) and 21 healthy donors (age range 24.0 ± 2.0 years, 12 males) were collected for 16S rRNA sequencing. No significant differences were observed in the alpha diversity indices between the two groups (Figure S1a). However, principal coordinate analysis (PCoA) representing beta diversity distances revealed significant variation in microbial structure between the MAFLD and healthy groups (*R* = 0.159, *p* = .003; Figure 1(a)). Additionally, patients with MAFLD exhibited significant alterations in the relative abundances of different types of bacteria at the genus level (Figure S1b). Specifically, there was a notable reduction in the relative abundance of several potentially beneficial bacteria, such as *Faecalibacterium*, *Lachnospiraceae NK4A136 group*, and *Ruminococcus*, and an obvious increase in the abundance of *Neisseria* (Figure 1(b) and Figure S1c), when compared to healthy controls. Further functional predictions based on the FAPROTAX database revealed seven distinct functional processes of the gut microbiota that differed between the two groups (Figure 1(c)). In addition, BugBase functional prediction revealed a significantly higher abundance of facultative anaerobic, gram-negative, and oxidative stress-tolerant microorganisms among patients with MAFLD (Figure 1(d)).

Similarly, no statistically significant differences in alpha diversity indices were observed between methionine- and choline-deficient (MCD) diet-induced MAFLD mice and healthy mice (Figure S1d). However, the PCoA plot revealed a gradual alteration in the gut microbiota composition of the mice as MAFLD progressed (Figure 1(e)). Consistent with human patients, the abundances of *Lachnospiraceae NK4A136 group* and *Ruminococcus* were similarly reduced in mice with MAFLD (Figure 1(f)). The findings demonstrated an altered composition and function of the gut microbiota in both MAFLD patients and mice, suggesting that manipulation of the gut microbiota was a promising strategy for the treatment of MAFLD.

### WMT significantly reduces liver fat accumulation in patients with MAFLD

**Figure 2.**
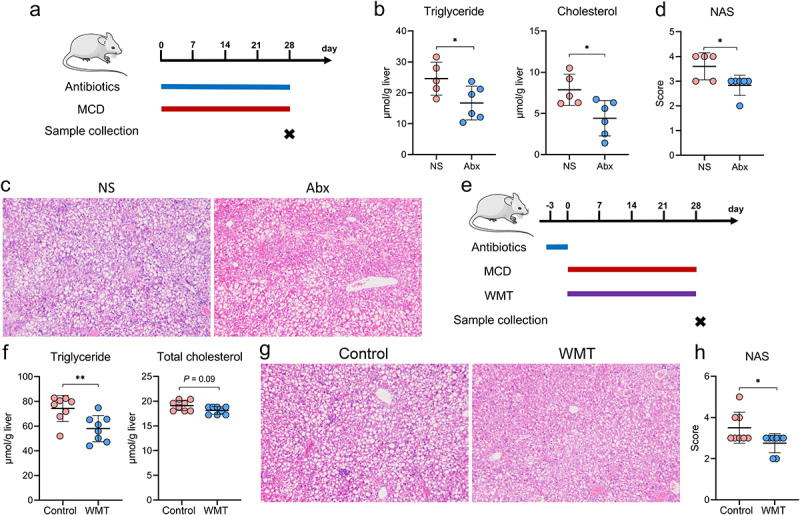
Administration of antibiotics or healthy microbiota significantly reduced hepatic fat accumulation in MAFLD mice (*n* = 5–6/group). (a) Study design involving MAFLD mice treated with antibiotics (Abx) or normal saline (NS). (b-d) Liver triglyceride and cholesterol levels, representative H&E staining of liver sections (magnification, ×100), and MAFLD activity score (NAS) in MAFLD mice treated with Abx or NS. (e) Study design involving MAFLD mice treated with MAFLD (control) or a healthy microbiota (WMT) (*n* = 8/group). (f-h) Levels of liver triglycerides and cholesterol, representative H&E staining of liver sections (magnification, ×100), and MAFLD activity score (NAS) in MAFLD mice treated with MAFLD or healthy microbiota. MAFLD, metabolic-associated fatty liver disease; MCD, methionine- and choline-deficient diet. *p < 0.05; **p < 0.01.

Consistent with previous studies,^17,23^ our findings demonstrated that targeting the gut microbiota with either an antibiotic (Abx) cocktail or WMT effectively decreased hepatic fat accumulation and liver injury in MAFLD mice (Figure 2). Given the potential adverse effects of excessive antibiotic use, including the development of multi-resistant bacteria, secondary infections, and systemic toxicity, our subsequent objective was to evaluate the therapeutic potential of WMT in reducing hepatic steatosis in patients with MAFLD.

**Figure 3.**
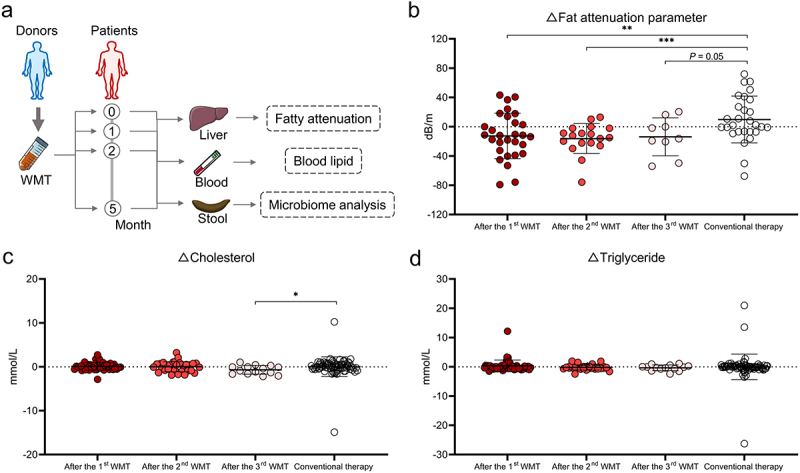
WMT significantly decreased liver fat accumulation in patients with MAFLD. (a) Study design for patients with MAFLD who received WMT. (b-d) Effects of WMT on fat attenuation parameters and blood triglyceride and cholesterol levels in patients with MAFLD. △MAFLD parameter = MAFLD parameter after treatment – MAFLD parameter at baseline. MAFLD, metabolic-associated fatty liver disease; WMT, washed microbiota transplantation. *p < 0.05; **p < 0.01.

Based on the inclusion and exclusion criteria, 59 patients with MAFLD underwent WMT, whereas 84 patients who received conventional therapy (lifestyle modification and/or lipid-lowering medications) were included in the cohort (Table S1 and Figure 3(a)). Of the 59 patients who underwent WMT, 17 successfully completed four treatment courses, 18 completed three courses, 21 completed two courses, and three managed to complete only one course. Figure 3(b) demonstrates a significant reduction in liver fat among patients with MAFLD who received WMT compared with those receiving conventional therapy. Following the second WMT, 42.9% (9/21) of the patients exhibited restoration of hepatic steatosis to the normal range, as evidenced by a fat attenuation parameter below 240 dB/m. Additionally, the blood lipid-lowering efficacy of WMT in patients with MAFLD was comparable to that of the conventional therapy (Figure 3(c-d)). Among the 167 WMT procedures in the cohort, three WMT-related AEs were observed. These included two cases of dizziness and one case of diarrhea, resulting in an overall incidence of 1.8%. Collectively, these findings indicated that WMT was an effective and safe therapeutic option for patients with MAFLD.

### WMT results in alterations in both the composition and function of the gut microbiota in patients with MAFLD

**Figure 4.**
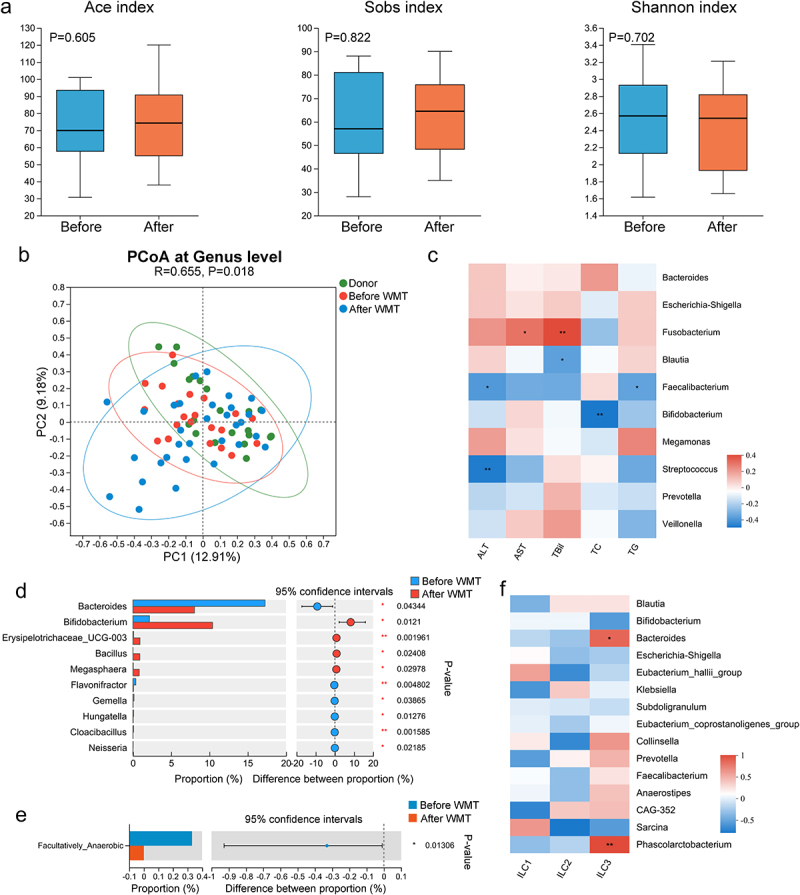
WMT altered the composition and function of gut microbiota in patients with MAFLD. (a) Box plots comparing the alpha diversity indices (Ace, Sobs, and Shannon) of the gut microbiota in patients with MAFLD before and after WMT. (b) PCoA plot illustrating microbial beta diversity among healthy donors and patients with MAFLD before and after WMT. (c) Heatmap depicting the correlations between genus-level abundance and levels of liver functional and blood lipid parameters in patients with MAFLD. (d-e) Bar plot showing the relative abundances of the top 10 differential genera and microbial function based on BugBase in patients with MAFLD before and after WMT. (f) Heatmap depicting correlations between genus-level abundance and proportions of circulating innate lymphoid cells in patients with MAFLD. MAFLD, metabolic-associated fatty liver disease; PCoA, principal coordinate analysis; WMT, washed microbiota transplantation. *p < 0.05; **p < 0.01; ***p < 0.001.

To elucidate the influence of WMT on the gut microbiota, stool samples were collected from patients with MAFLD pre- and post-WMT and analyzed using 16S rRNA sequencing. Notably, the gut microbial composition of patients with MAFLD changed significantly after undergoing WMT, despite the absence of significant alterations in alpha diversity indices (Figure 4(a-b)). Moreover, a negative correlation was observed between the relative abundances of potentially beneficial genera, such as *Faecalibacterium* and *Bifidobacterium*, and the disease severity parameters (Figure 4(c)). Additionally, the abundance of *Neisseria* decreased significantly following WMT and was significantly elevated in patients with MAFLD. In contrast, the abundance of beneficial genera, including *Bifidobacterium* and *Megasphaera*, increased significantly after WMT (Figure 4(d) and Figure S2). BugBase algorithm-based prediction indicated that WMT resulted in a significant reduction in facultative anaerobic microorganisms in patients with MAFLD (Figure 4(e)). These findings suggested that WMT resulted in modifications in the gut microbial composition, with an increase in the abundance of beneficial genera and a decrease in the abundance of pathogenic bacteria.

Given the well-established interaction between ILC3s and gut microbiota,^18,24^ the relationship between the abundance of gut microbiota and proportion of circulating ILC3s was explored. The correlation heatmap depicted a positive correlation between circulating ILC3 levels and both *Bacteroides* and *Phascolarctobacterium* abundance in patients with MAFLD (Figure 4(f)). However, the relationship between ILC3s and MAFLD, as well as the potential regulatory effect of WMT on ILC3s, remained unexplored.

### The proportion of ILC3s increases outside the liver and decreases within the liver in human and murine MAFLD

**Figure 5.**
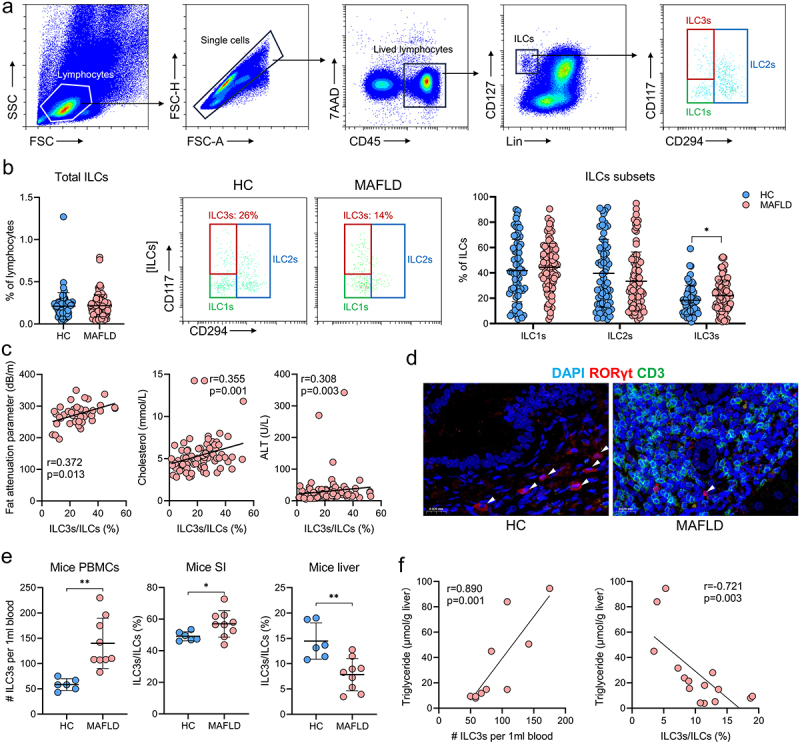
The proportion of ILC3s increased outside the liver and decreased within the liver in both human and murine MAFLD. (a) Representative flow cytometric gating strategy. (b) Comparison of the proportions of circulating total ILCs and ILCs subsets between healthy controls (HC, *n* = 66) and patients with MAFLD (*n* = 93). (c) Correlation between the proportion of circulating ILC3s and the levels of fat attenuation parameters, blood cholesterol, LDL-C, and ALT in patients with MAFLD. (d) Representative confocal microscopy images of liver sections from patients with or without MAFLD; arrows indicate ILC3s (CD3^−^RORγt^+^); scale bar: 20 μm. (e) Comparison of ILC3 levels in PBMCs, small intestine (SI), and liver between healthy (*n* = 6) and MAFLD mice (*n* = 9). (f) Correlation between the levels of circulating and hepatic ILC3s and hepatic triglyceride concentrations in mice. ILC3, group 3 innate lymphoid cells; ILCs, innate lymphoid cells; MAFLD, metabolic-associated fatty liver disease. *p < 0.05; **p < 0.01.

To investigate the relationship between ILC3s and MAFLD, flow cytometric analyses were conducted on whole blood samples collected from 93 patients with MAFLD (mean age: 62.0, range: 49.0–70.5, 49 males) and 66 individuals without MAFLD (mean age: 41.5, range: 32.8–53.0, 41 males). The gating strategy for analyzing total ILCs and their subsets in human peripheral blood mononuclear cells (PBMCs) is shown in Figure 5(a). The proportions of total ILCs, ILC1s, and ILC2s in PBMCs were similar between the two groups. However, patients with MAFLD displayed a significantly increased frequency of ILC3s among total ILCs compared to those without MAFLD (Figure 5(b)). Moreover, subsequent correlation analysis revealed a significant positive correlation between the frequency of circulating ILC3s and various disease severity indicators, such as the fat attenuation parameter, serum cholesterol, low-density lipoprotein cholesterol (LDL-C), and alanine transaminase (ALT) (Figure 5(c)). Contrary to the results of ILC3s in the peripheral blood, immunofluorescence results suggested that the levels of ILC3s in the liver of patients with MAFLD were significantly lower than those without MAFLD (Figure 5(d)).

Consistent with the findings observed in human PBMCs, the levels of ILC3s in the PBMCs of mice with MAFLD were significantly elevated compared to those in healthy mice (Figure 5(e)). In addition, mice with MAFLD exhibited an altered distribution of ILC3s, with a higher proportion in the small intestine and lower proportion in the liver (Figure 5(e)). Furthermore, the concentration of hepatic triglycerides was positively correlated with the levels of circulating ILC3s and negatively correlated with the proportion of hepatic ILC3s (Figure 5(f)). These results indicated that measuring both circulating and hepatic ILC3s could be used as a strategy of evaluating the severity of MAFLD. Additionally, targeting hepatic ILC3s may be a promising therapeutic approach for MAFLD.

### WMT significantly decreases the proportion of circulating ILC3s and increases the proportion of hepatic ILC3s in MAFLD patients and mice

**Figure 6.**
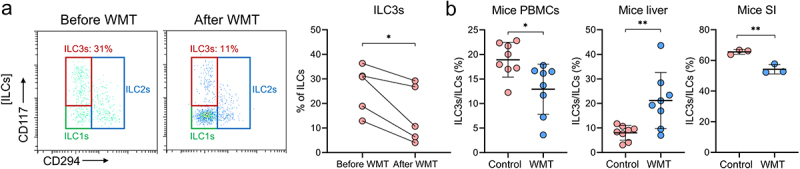
WMT significantly decreased the proportion of circulating ILC3s in MAFLD patients and mice, and increased the proportion of hepatic ILC3s in MAFLD mice. (A) The proportion of circulating ILC3s in patients with MAFLD before and after WMT (*n* = 5). (B) Proportion of ILC3s from PBMCs, liver, and small intestine (*n* = 3) in MAFLD mice treated with MAFLD (control) or healthy microbiota (WMT). *n* = 8 mice per group. ILC3, type 3 innate lymphoid cells; MAFLD, metabolic-associated fatty liver disease; WMT, washed microbiota transplantation. *p < 0.05; **p < 0.01.

Next, we investigated the influence of WMT on ILC3 levels in MAFLD patients and mice. A significant decrease in the proportion of circulating ILC3s was observed in patients with MAFLD after WMT (Figure 6(a)). This finding was supported by additional evidence from murine MAFLD models (Figure 6(b)). Moreover, WMT resulted in a significant increase in hepatic ILC3s and a decrease in small intestinal ILC3s in mice with MAFLD (Figure 6(b)). Based on the findings of a recent study reporting the potential of FMT to mitigate intestinal graft-versus-host disease by restoring ILC3 levels in the affected region,^22^ we hypothesized that WMT could similarly alleviate MAFLD by increasing hepatic ILC3 proportions.

### WMT promotes ILC3 homing to the liver through the upregulation of CXCR6 expression on ILC3s

**Figure 7.**
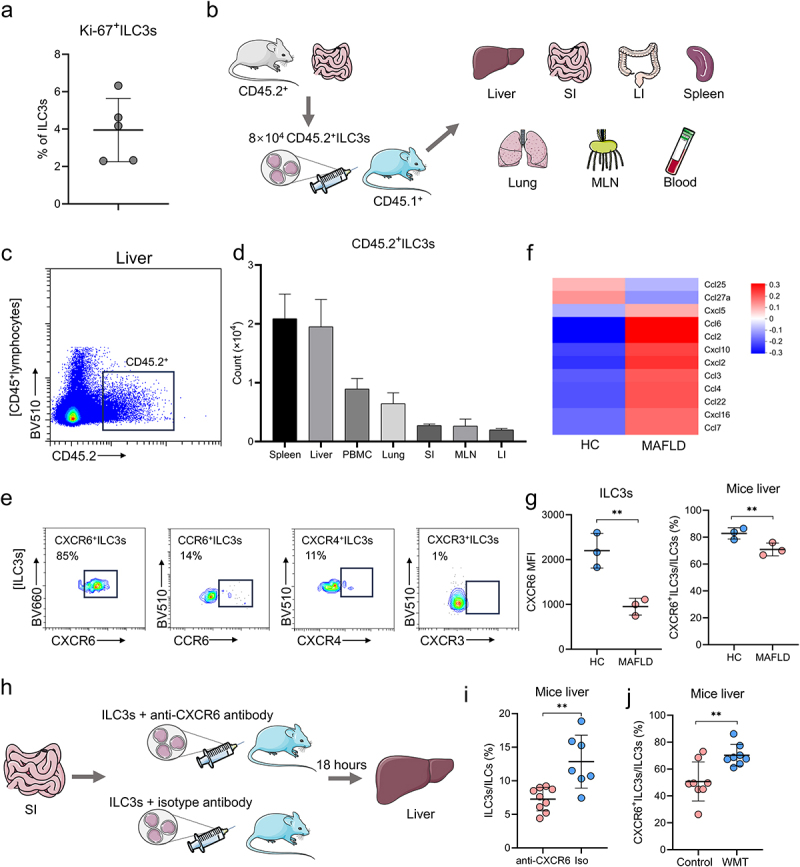
WMT might promote ILC3s homing to the liver through the upregulation of CXCR6 expression on ILC3s. (a) Proportion of Ki-67^+^ ILC3s in the liver of MAFLD mice after WMT. (b) Study design for intestine-derived ILC3s tracing experiments. (c) Representative flow plot depicting intestine-derived CD45.2^+^ ILC3s in the livers of CD45.1^+^ MAFLD mice (*n* = 3/group). (d) The number of CD45.2^+^ ILC3s migrating to the spleen, liver, PBMC, small intestine (SI), mesenteric lymph node (MLN), and large intestine (LI) of CD45.1^+^ MAFLD mice. (e) Representative flow plots depicting the expression of different liver-homing chemokine receptors in hepatic ILC3s of healthy mice. (f) Heatmap of RNA sequencing data showing differentially expressed chemokine genes in healthy and MAFLD mice. (g) Comparison of the mean fluorescence intensity (MFI) of CXCR6 on ILC3s and the proportion of CXCR6^+^ ILC3s in the liver between healthy and MAFLD mice (*n* = 3/group). (h) Study design of CXCR6 blocking experiments. (i) The proportion of ILC3s in the livers of MAFLD mice after receiving ILC3s treated with CXCR6 antibody (*n* = 9) or isotype control (*n* = 7). (j) The proportion of CXCR6^+^ ILC3s in the livers of mice treated with MAFLD (control, *n* = 8) or healthy microbiota (WMT, *n* = 8). MAFLD, metabolic-associated fatty liver disease. *p < 0.05; **p < 0.01.

Next, we elucidated the primary cause of increased hepatic ILC3s following WMT. Despite the high proliferation of ILC3s in the murine intestine,^18^ the low positive rate of Ki-67 in hepatic ILC3s following WMT suggested that the increase in ILC3s might not be attributed to their proliferation (Figure 7(a)).

Following WMT treatment, a notable decrease in the proportion of ILC3s was observed in the intestine and PBMCs, whereas an increased proportion was observed in the liver (Figure 6(b)). This suggested that the increased hepatic ILC3s might have migrated from other sites in response to WMT treatment. Furthermore, as the lamina propria of the small intestine has been identified as the primary habitat for ILC3s,^25^ we hypothesized that the observed increase in hepatic ILC3s following WMT could be attributed to their migration from the intestine. Additionally, previous studies have shown that ILC2s originating from the small intestine migrate toward the lungs in response to pulmonary infection.^26^ Consequently, we speculated that the observed increase in hepatic ILC3s following WMT could be attributed to their migration from the intestine to support the immune response in the liver.

To investigate the migratory preferences of intestinal ILC3s toward the liver, we intravenously injected intestine-derived ILC3s from healthy CD45.2^+^ mice into the tail vein of CD45.1^+^ MAFLD mice (Figure 7(b)). After 18 h of injection, a significant number of intestine-derived CD45.2^+^ ILC3s migrated to the livers of CD45.1^+^ MAFLD mice, constituting approximately 25% of the injected ILC3s (Figure 7(c-d)). This finding indicated a strong affinity of intestinal ILC3s for the liver.

Chemokines and their corresponding receptors play crucial roles in facilitating immune cell migration. Specifically, CXCL16/CXCR6, CXCL12/CXCR4, CXCL10/CXCR3, and CCL20/CCR6 have been identified as key players in immune cell homing to the liver.^27,28^ Flow cytometric analysis revealed that hepatic ILC3s expressed the highest levels of liver-homing chemokine receptor CXCR6, with a positivity rate of 85% (Figure 7(e)). mRNA sequencing analysis revealed a significant upregulation of CXCL16 mRNA expression in the livers of MAFLD mice (Figure 7(f)). However, flow cytometry results demonstrated a significant reduction in the mean fluorescence intensity (MFI) of CXCR6 on ILC3s and the proportion of CXCR6^+^ ILC3s in the livers of MAFLD mice (Figure 7(g)). These findings may explain the decrease in hepatic ILC3s in MAFLD mice.

To investigate the impact of the CXCL16/CXCR6 axis on ILC3 migration to the liver, MAFLD mice were administered ILC3s pre-treated with either an anti-CXCR6 antibody or an isotype control (Figure 7(h)). As expected, mice that received ILC3s pre-treated with the anti-CXCR6 antibody exhibited significantly reduced levels of hepatic ILC3s compared to those that received ILC3s pre-treated with the isotype control (Figure 7(i)). Similarly, blocking CXCL16 with a neutralizing antibody inhibited the migration of ILC3s into the liver (Figure S3). These findings validated the involvement of the CXCL16/CXCR6 axis in ILC3 migration to the liver. In addition, the present study observed a significant increase in the expression of CXCR6 on hepatic ILC3s in MAFLD mice following WMT (Figure 7(j)). These findings collectively suggested that WMT potentially facilitated the migration of ILC3s to the liver by upregulating the expression of CXCR6 on ILC3s.

### Hepatic ILC3s mitigate hepatic steatosis and liver damage via the secretion of IL-22

**Figure 8.**
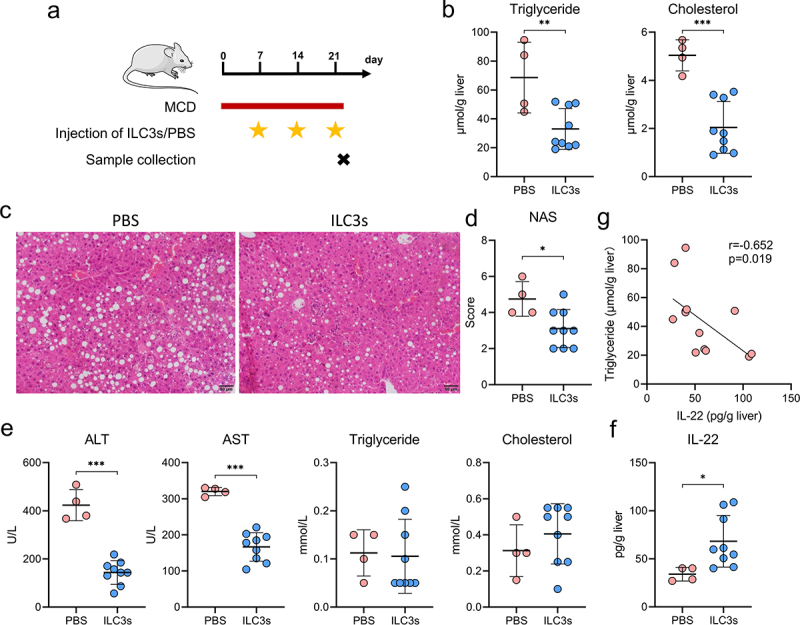
Hepatic ILC3s mitigated hepatic steatosis and liver damage via IL-22 secretion. (a) Study design involving MAFLD mice treated with PBS (*n* = 4) or ILC3s (*n* = 9). (b) Concentrations of triglycerides and cholesterol in the liver of MAFLD mice. (c) Representative H&E staining of the liver sections; scale bar: 50 μm. (d-e) MAFLD activity score (NAS) and levels of blood alanine transaminase (ALT), aspartate transaminase (AST), triglyceride, and cholesterol in MAFLD mice treated with ILC3s or PBS. (f) IL-22 concentration in the livers of MAFLD mice treated with ILC3s or PBS. (g) Correlation between the concentration of hepatic IL-22 and triglyceride levels in mice. ILC3, group 3 innate lymphoid cells; MAFLD, metabolic-associated fatty liver disease; MCD, methionine- and choline-deficient diet. *p < 0.05; **p < 0.01; ***p < 0.001.

To investigate the role of hepatic ILC3s in the development of MAFLD, an adoptive transfer of intestinal ILC3s into MAFLD mice was conducted because intestinal ILC3s tend to migrate to the liver (Figure 8(a)). Following weekly administration of ILC3s for three consecutive weeks, a significant decrease in the concentration of hepatic triglycerides and cholesterol, a reduction in the MAFLD activity score, and a decline in the plasma concentrations of alanine transaminase and aspartate transaminase were observed in mice with MAFLD (Figure 8(b-e)).

Given the demonstrated proficiency of ILC3s in cytokine production,^25,29^ the levels of classical cytokines, including IL-2, IL-17A, IL-22, granulocyte-macrophage colony stimulating factor (GM-CSF), and lymphotoxin, were assessed in the livers of MAFLD mice. Following the adoptive transfer of ILC3s, MAFLD mice exhibited a significant increase in liver IL-22 levels compared to those treated with PBS (Figure 8(f)). However, no significant differences were observed in the IL-2, IL-17A, GM-CSF, or lymphotoxin levels (Figure S4). Furthermore, a negative correlation was established between IL-22 concentrations and liver triglyceride levels in MAFLD mice (Figure 8(g)). These findings collectively suggest that hepatic ILC3s exert a protective effect against hepatic steatosis and liver damage via the secretion of IL-22.

## Discussion

**Figure 9.**
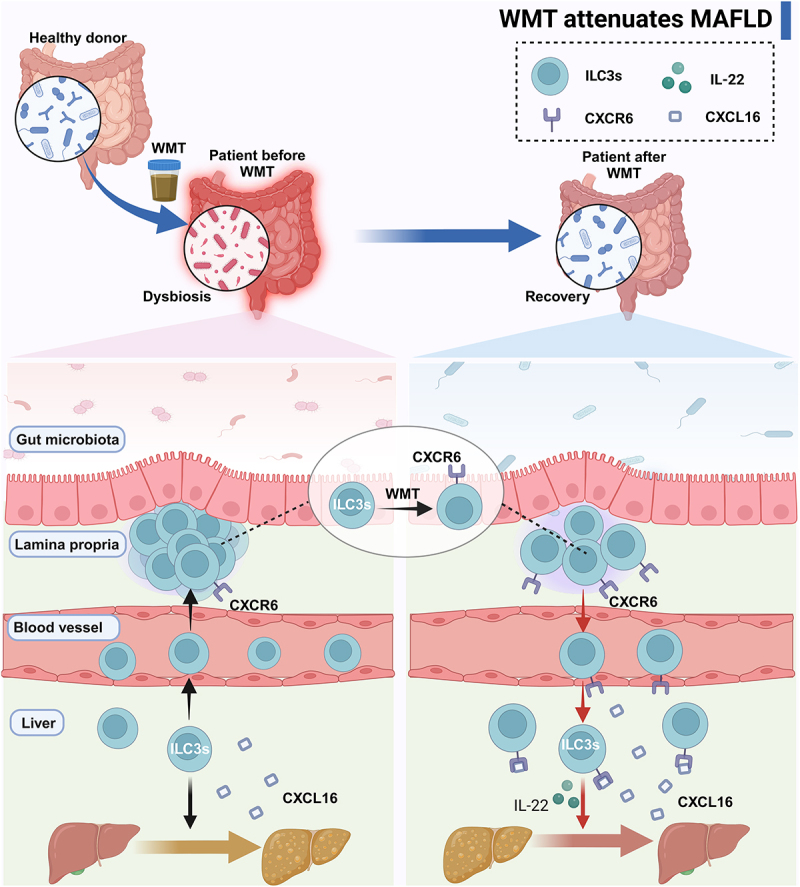
Graphical abstract. MAFLD patients and mice presented an altered composition of gut microbiota and an abnormal distribution of ILC3s due to the reduced expression of CXCR6 on ILC3s. WMT treatment resulted in the reconfiguration of the gut microbiota composition and upregulation of CXCR6 expression on ILC3s, consequently leading to liver-homing of ILC3s from ex-hepatic sites. Hepatic ILC3s attenuated hepatic steatosis in MAFLD patients and mouse models by secreting IL-22. ILC3s, group 3 innate lymphoid cells; MAFLD, metabolic-associated fatty liver disease; WMT, washed microbiota transplantation.

In the present study, we observed that patients with MAFLD exhibited an altered gut microbiota composition characterized by reduced levels of beneficial bacteria and an increased abundance of pathogenic bacteria. Furthermore, the administration of WMT was found to induce alterations in the gut microbiota composition, leading to an increase in the abundance of beneficial genera and a decrease in the abundance of pathogenic bacteria. This consequently resulted in a decrease in hepatic steatosis in both MAFLD patients and mice. Additionally, we observed a decrease in the expression of the liver-homing chemokine receptor CXCR6 on ILC3s in human and murine MAFLD, leading to an atypical distribution of ILC3s, characterized by a significant reduction in ILC3s in the liver and an increase in ILC3s outside the liver. Moreover, the severity of the disease exhibited a negative correlation with the proportion of hepatic ILC3s. These ILC3s demonstrated a mitigating effect on hepatic steatosis *via* the secretion of IL-22. Mechanistically, WMT upregulated CXCR6 expression on ILC3s, facilitating their migration to the liver of MAFLD mice *via* the CXCL16/CXCR6 axis. This ultimately improved the MAFLD (Figure 9).

Our study aligns with previous studies that have reported variations in gut microbiota composition between patients with MAFLD and healthy individuals.^7,8^ Additionally, our findings revealed a significant decline in the abundance of beneficial bacteria, including *Faecalibacterium*, *Lachnospiraceae NK4A136 group*, and *Ruminococcus*, as previously documented.^8^ Conversely, there was a marked increase in the abundance of pathogenic bacteria, specifically *Neisseria*, in patients with MAFLD. Following the administration of WMT to regulate the gut microbiota, significant alterations occurred in the gut microbiota composition in patients with MAFLD. These alterations included a notable increase in the abundance of beneficial genera such as *Bifidobacterium* and *Megasphaera* and a decrease in the abundance of *Neisseria*. These findings suggest that WMT could partially restore the balance of the gut microbiota and serve as a potential therapeutic approach for MAFLD.

In the present study, bacterial functional analysis revealed a marked reduction in facultative anaerobes in patients with MAFLD following WMT treatment. Notably, the abundance of two facultative anaerobes, *Gemella* and *Neisseria*, significantly increased in patients and decreased after WMT treatment. However, the abundance of these two genera was not significantly correlated with the clinical markers of patients. Therefore, further investigation is necessary to determine the potential involvement of these bacteria in the progression of MAFLD and their impact on the efficacy of WMT. Additionally, the abundance of *Bacteroides* in MAFLD patients was positively correlated with the proportion of ILC3s in the peripheral blood and was significantly reduced after WMT. This indicates that *Bacteroides* may play a pivotal role in modulating the levels of peripheral blood ILC3s via WMT. However, this hypothesis needs to be validated in further experiments.

Previous investigations have established the beneficial effects of FMT on various aspects of NAFLD, including improvements in small intestinal permeability, alterations in gut microbiota composition, modifications in hepatic DNA methylation profiles, and reduction in hepatic fat accumulation. ^30–32^ However, there is currently a lack of research on the efficacy of WMT in the treatment of MAFLD. In this study, we report that WMT effectively reduced hepatic steatosis in both MAFLD mice and patients. Furthermore, the incidence of WMT-related AEs was 1.8% and all AEs were mild, transient, and self-limiting. Collectively, these findings suggest that WMT is a safe and effective therapeutic option for MAFLD.

Previous studies have implicated ILC3s in various metabolic and chronic liver diseases.^21,33,34^ In patients with type 2 diabetes, the frequency of peripheral ILC3s is significantly lower than that in healthy individuals, and there is a negative correlation between the levels of fasting glucose and glycated hemoglobin and the frequency of peripheral ILC3s.^34^ In contrast, our previous study revealed that patients with hyperuricemia present significantly higher levels of ILC3s in PBMCs, which are positively correlated with disease severity.^33^ Moreover, Wang et al. observed a higher prevalence of circulating ILC3s among individuals with cirrhosis, a trend that further exacerbates as the disease advances.^21^ In the present study, abnormal distribution of ILC3s was observed in human and murine MAFLD. Specifically, a decrease in hepatic ILC3s and an increase in ILC3s in PBMCs and small intestine were observed. Additionally, a positive correlation was identified between hepatic triglyceride concentration and peripheral ILC3 levels, whereas a negative correlation was observed between the hepatic ILC3 proportion and triglyceride levels. These findings suggest that circulating and hepatic ILC3s may serve as biomarkers for assessing MAFLD severity.

ILC3s, a subset of immune cells regulated by gut microbiota,^18^ have emerged as potential targets for FMT-based therapeutic interventions.^22^ Our study revealed a significant increase in the abundance of hepatic ILC3s in MAFLD mice after WMT treatment. Contrary to the conventional perception of ILC3s as tissue-resident cells with high proliferative potential under inflammatory conditions,^35,36^ our findings revealed a relatively low (~4%) proliferation rate of hepatic ILC3s in mice subjected to WMT treatment. Meanwhile, a significant decrease in the proportion of ex-hepatic ILC3s was observed following WMT, suggesting potential enhancement of ex-hepatic ILC3 migration to the liver. Despite a significant reduction in the proportion of ILC3s in PBMCs of both MAFLD patients and mice following WMT, the presence of ILC3s in the circulatory system remains minimal.^37^ In addition, studies have established that the intestine serves as the primary site of ILC3s.^38^ Recent studies have also revealed the migratory capacity of intestinal ILC3s toward the kidneys.^39^ Similarly, our findings also indicate that intestinal ILC3s have a strong tendency of homing to the liver. This finding implies that the increased presence of ILC3s in the liver after WMT is likely due to their migration from the intestine.

The chemokine-chemokine receptor axis is a vital group of chemoattractant signals that facilitates the targeted migration of immune cells.^40^ Consistent with previous findings,^41^ our investigation revealed significant expression of CXCR6, a liver homing-chemokine receptor, on ILC3s in both the intestine and liver of mice. A previous study indicated that CXCR6 is responsible for guiding NK cells, a representative member of the ILC family, to the liver.^42,43^ Furthermore, our study revealed that blocking the CXCL16/CXCR6 chemotaxis pathway using CXCL16 or CXCR6 antibodies effectively impedes the migration of ILC3s to the liver. Additionally, hepatic ILC3s in mice with MAFLD exhibited a significant reduction in CXCR6 expression, potentially contributing to the migration of ILC3s from the liver to the small intestine and PBMCs. However, hepatic ILC3s in MAFLD mice exhibited elevated CXCR6 expression following WMT, suggesting that WMT could enhance CXCR6 expression on ILC3s and subsequently induce their homing to the liver in MAFLD mice.

A recent study reported that knockout of whole-body ILC3s with the RORγt antagonist A213 in Rag2 KO mice resulted in the development of fatty liver caused by a high-fat diet.^44^ However, the effect of hepatic ILC3s on fatty liver disease remains unclear. Considering the liver-homing tendency of intestinal ILC3s in our study, intestine-derived ILC3s were adoptively transferred into MAFLD mice to increase the number of hepatic ILC3s. This augmentation of hepatic ILC3s resulted in a notable reduction in hepatic steatosis, amelioration of pathological injury, and a decline in liver enzyme levels, thereby suggesting a protective function for hepatic ILC3s in MAFLD. Intestinal CXCR6^+^ ILC3s are a significant subset of ILC3s that produce IL-22.^45^ In MAFLD mice, hepatic ILC3s migrate from the intestine to the liver through the CXCL16/CXCR6 chemotaxis axis, resulting in increased IL-22 levels in the liver. This increase in IL-22 levels has been shown to mitigate hepatic steatosis.^46^ These findings suggest that hepatic ILC3s play a protective role against MAFLD by secreting the hepatoprotective cytokine, IL-22. Consequently, targeting liver-homing ILC3s may be a promising therapeutic strategy for MAFLD treatment.

This study had several limitations that warrant acknowledgment. First, the retrospective design of the study did not permit capture of all confounding variables, such as diet and exercise, including type, frequency, intensity, and duration, which could potentially influence MAFLD. Furthermore, missing clinical data could not be collected. Second, the migratory nature of hepatic ILC3s in mice with MAFLD presents a challenge, specifically in targeting and knocking out these cells. Third, only the MCD-induced fatty liver mouse model was used in the present study, whereas high-fat diet-induced models were not used. Fourth, blood samples from only five MAFLD patients receiving WMT were collected to study the effect of WMT on peripheral blood ILC3s. Thus, our findings remain to be confirmed in studies with larger sample sizes. Finally, further investigation is necessary to identify the specific key species involved and the mechanism responsible for the increased expression of CXCR6 on ILC3s.

Collectively, both MAFLD patients and mice exhibited alterations in the gut microbiota composition and distribution of ILC3s, owing to the reduced CXCR6 expression on ILC3s. WMT treatment resulted in reconfiguration of gut microbiota composition and upregulation of CXCR6 expression on ILC3s. Consequently, this leads to liver homing of ILC3s from the ex-hepatic sites. Moreover, hepatic ILC3s attenuated hepatic steatosis in both MAFLD patients and mice by secreting IL-22. These findings highlight that WMT and targeting of liver-homing ILC3s could be promising strategies for the treatment of MAFLD.

## Patients and methods

### Patient selection and sample collection

This retrospective study included hospitalized patients aged ≥18 years who presented with MAFLD at the First Affiliated Hospital of Guangdong Pharmaceutical University between April 2017 and October 2022. The enrolled patients were assigned to either the WMT or the control group based on their treatment status. Patients with incomplete clinical data or a lack of follow-up were excluded. In the WMT group, patients receiving lipid-lowering medications such as metformin and statins were also excluded. Patients in the control group received conventional therapy, including lifestyle modifications and/or lipid-lowering medications. MAFLD was diagnosed as previously described.^47^

Whole blood and stool samples were collected from patients diagnosed with MAFLD prior to each WMT procedure. Whole blood was used for the quantification of ILCs. Stool samples were stored at −80°C until 16S rRNA gene sequencing.

The study was approved by the Ethics Committee of the First Affiliated Hospital of Guangdong Pharmaceutical University (#2021–13). Written informed consent was obtained from all the patients.

### Data collection

Clinical data were collected from patient medical records, including information on sex, age, body mass index (BMI), medication usage, FibroTouch fat attenuation parameters, and laboratory test results for triglyceride and total cholesterol levels.

### WMT procedure

Healthy donor screening, washed microbiota suspension preparation, and microbiota transplantation were conducted following previously established protocols.^48^ A questionnaire assessing medical history, fecal examination for pathogens, and blood tests was used to screen potential donors. The bacterial suspension was prepared in a Biosafety Level 2 laboratory with the assistance of skilled professionals who exclusively used disposable sterile materials. To prepare 100 g of fecal samples from healthy donors, the samples were mixed with 500 mL of 0.9% saline and subsequently microfiltered using an automatic purification device (GenFMTer, FMT Medical). The microbial pellet was washed three times, followed by the addition of 100 mL saline solution to prepare the bacterial suspensions. The suspension was maintained at a constant temperature of 37°C in a water bath and infused within one hour to prevent contamination or composition changes. Furthermore, a 2 mL sample of the bacterial suspension was stored at −80°C for future infectious pathogen testing and traceability analysis. A total of 120 mL of microbial suspension was transplanted into patients through a nasojejunal or colonic transendoscopic enteral tube daily for 3 consecutive days.

### Animals

C57BL/6 CD45.1 congenic and wild-type (CD45.2) mice were purchased from Jackson Laboratory and Guangdong Medical Laboratory Animal Center, respectively. The mice were housed in temperature- and light-controlled animal quarters with ad libitum access to chow and water. Prior to commencing the experiments, the mice were subjected to adaptive feeding for a minimum duration of one week. Animal experiments were approved by the Animal Ethics Committee of the First Affiliated Hospital of Guangdong Pharmaceutical University (#2022–14) and conducted following the Animals in Research: Reporting In Vivo Experiments guideline.

To induce MAFLD, a methionine- and choline-deficient (MCD) diet was fed to 14-week-old male mice for 4 weeks.^49^ Stool samples were obtained from three groups of mice: healthy mice, mice fed MCD for 2 weeks (MAFLD-2W), and mice fed MCD for 4 weeks (MAFLD-4W). All samples were promptly stored at −80°C until further processing. Blood and liver samples were collected from both healthy and MAFLD mice for ILC3 detection, liver triglyceride quantification, histological evaluation, and mRNA sequencing.

### Cell isolation

Peripheral blood mononuclear cells (PMBCs) were isolated from the blood samples using Ficoll gradient centrifugation.

Intestinal tissues from the mice were harvested and rinsed with PBS to remove fecal content. After removing the mesenteric adipose and Peyer’s patches, the intestine was longitudinally opened and cut into 1 cm segments. The tissue was incubated in an extraction solution (RPMI-1640, 2% FBS, 10 mM DTT, and 1 mM EDTA) at 37°C with shaking at 180 rpm for 15 min. The remaining tissues were washed in PBS, cut into 1 mm pieces, and incubated in digestion solution (RPMI-1640, 2% FBS, 1 mg/mL collagenase type II, and 0.5 mg/mL Dispase) at 37°C with continuous shaking at 180 rpm for 45 min. Following digestion, the remaining tissues were filtered through a 70 μm strainer. The supernatant was centrifuged to isolate lamina propria leukocytes.

Liver tissues were mechanically disrupted by passing through a 70 μm strainer in PBS. The resulting suspension was centrifuged at 1500 rpm for 5 min at 4°C. After discarding the supernatant, the pellet was resuspended in 3 mL 40% Percoll. This mixture was then layered on top of 4 mL of 100% Percoll and centrifuged at 800 ×g for 20 min at 4°C, without acceleration or braking. The interphase layer was collected, washed with PBS, centrifuged, and resuspended in PBS.

The spleen and mesenteric lymph nodes were mechanically disrupted using a 70 μm strainer in PBS. The resulting cell suspension was centrifuged at 1500 rpm for 5 min at 4°C. The supernatant was then discarded.

Lung tissue was harvested, sectioned into 1 mm fragments, and incubated in digestion solution (RPMI-1640, 2% FBS, 1 mg/mL collagenase type II, and 0.5 mg/mL Dispase) at 37°C with continuous shaking at 180 rpm for 45 min. The digested tissues were filtered through a 70 μm strainer. The supernatant was centrifuged to isolate lung leukocytes.

### Flow cytometry and cell sorting

ILC subsets in human PBMCs were characterized by staining PBMCs with 7-Amino-actinomycin D and specific antibodies. The following antibodies were used in this study: anti-CD3 (HIT3a), anti-CD5 (UCHT2), anti-CD11c (3.9), anti-CD16 (B73.1), anti-CD19 (HIB19), anti-TCR α/β (IP26), anti-CD117 (A3C6E2), anti-CD127 (A019D5), anti-CD294 (BM16), and anti-CD45 (HI30; all from BioLegend).

To characterize or sort ILC3s of mice, cell suspensions were stained with the following antibodies: anti-CD3ε (145-2C11), anti-CD19 (6D5), anti-CD11c (N418), anti-CD11b (M1/70), anti-CD5 (53–7.3), anti-TER-119 (TER-119), anti-CD117 (2B8), anti-CD45.2 (104), anti-CCR6 (29-2L17), anti-CXCR3 (CXCR3–173), anti-CXCR4 (L276F12), anti-CXCR6 (SA051D1), anti-Ki-67 (16A8), anti-CD45 (I3/2.3), anti-CD45.1 (A20), anti-CD127 (A7R34; all from BioLegend), and anti-RORγt (AFKJS-9; eBioscience). Intranuclear staining was performed using a Foxp3 staining kit (eBioscience) following the manufacturer’s instructions.

The stained cells were analyzed using a CytoFlex flow cytometer (Beckman Coulter) or purified using a BD Influx cell sorter (BD Biosciences). Data analysis was performed using the CytExpert (Beckman Coulter) software.

### Antibiotic treatment experiments in mice

Two groups of 14-week-old male mice were subjected to dietary intervention using an MCD diet. Additionally, one group received a daily oral gavage of a 0.2 mL antibiotic cocktail, while the other group received normal saline. This intervention was carried out for four weeks. The antibiotic cocktail comprised ampicillin (1 g/L), neomycin (1 g/L), metronidazole (1 g/L), and vancomycin hydrochloride (0.5 g/L).^50^ Liver tissues were collected to quantify liver fat and to conduct histological evaluations.

### WMT experiments in MAFLD mice

Mice were subjected to a three-day regimen of antibiotic cocktail administration. Following this, the mice were fed with MCD diet and randomly divided into two groups (*n* = 8/group): (1) WMT group, administered 0.2 mL gut microbial suspension from healthy mice; (2) Control group, administered 0.2 mL gut microbial suspension from MAFLD mice. Fecal samples were collected from both healthy and MAFLD mice using sterile Eppendorf tubes. Subsequently, the samples (0.1 g) were suspended in 1 mL normal saline for approximately 10 min, followed by homogenization and centrifugation at 1000 rpm for 3 min. The resulting suspension was centrifuged at 8000 rpm for 5 min, followed by two washes of the fecal pellet using normal saline.^51^ The supernatant was discarded, and 1 mL of saline was added to the microbiota pellet obtained from the 0.1 g of feces to prepare bacterial suspensions. The microbial suspension was administered by oral gavage daily for four weeks. Thereafter, the liver tissues were collected for liver fat quantification, histological evaluation, and ILC3s detection. Blood samples were collected for ILC3 measurements.

### Adoptive transfer experiments

Approximately 7 × 10^4^ highly purified ILC3s or PBS were injected intravenously into the tail vein of MAFLD mice once per week for three consecutive weeks. Mice were euthanized 24 h after the last injection. Liver tissues were collected for fat quantification and histological evaluation.

### Intestine-derived ILC3 tracing experiments

Approximately 8 × 10^4^ highly purified ILC3s were sorted from the intestines of C57BL/6 wild-type (CD45.2) mice. These cells were then injected intravenously into the tail vein of MAFLD mice (C57BL/6 CD45.1 congenic mice). Recipient mice were euthanized 18 h post-injection. Tissue samples from various organs, including the liver, small intestine, large intestine, spleen, lungs, mesenteric lymph nodes, and blood, were collected to measure the levels of intestine-derived CD45.2^+^ ILC3s.

### CXCR6 blocking experiments

Approximately 1 × 10^5^ ILC3s were incubated with CXCR6 antibody (MAB2145, R&D Systems) or isotype control (MAB0061, R&D Systems) (10 μg/mL) for 30 min. Subsequently, the treated ILC3s were intravenously injected into the tail vein of MAFLD mice. The recipient mice were euthanized 18 h post-injection. Liver tissue samples were collected to measure ILC3 levels.

### CXCL16 blocking experiments

To block CXCL16, MAFLD mice were intraperitoneally injected with rabbit anti-mouse CXCL16-neutralizing antibody (100 μg; 50142-T48, Sino Biological). Control mice were administered 100 μg of isotype IgG (CR1, Sino Biological). Two days later, approximately 2 × 10^4^ ILC3s were intravenously injected into the tail veins of MAFLD mice. Recipient mice were euthanized 18 h post-injection. Liver tissue samples were collected to measure the ILC3 levels.

### 16S rRNA gene sequencing and data analysis

Microbiome 16S rRNA gene sequencing was performed at Majorbio Co. Ltd. (Shanghai, China), following an established protocol.^52^ Microbial genomic DNA was extracted from the fecal sample (0.1 g) using an E.Z.N.A.® soil DNA kit (Omega Bio-tek), following the manufacturer’s protocol. The V3-V4 region of the bacterial 16S rRNA gene was amplified using PCR with the forward primer 338F and reverse primer 806 R. The amplicons were visualized by agarose gel electrophoresis. To monitor contamination, amplification of the PCR blank controls was performed. Subsequently, the amplicons were sequenced on a MiSeq platform (Illumina). Microbial sequencing data were deposited in the Sequence Read Archive database under PRJNA number 1,035,094.

Read pairs were demultiplexed, merged using FLASH (version 1.2.11),^53^ and filtered using fastp (version 0.19.6).^54^ Briefly, reads shorter than 10 bp and those with a base quality score below Phred 20 were eliminated. Subsequently, paired reads with a minimum overlap of 10 bp were merged into single sequences, applying a maximum mismatch ratio of 0.2 to filter out non-conforming sequences. Samples were differentiated and sequence orientation was adjusted based on the barcodes and primers at both ends of the sequence. The stringent tolerance for barcode mismatches was set at 0, whereas a more lenient threshold for primer mismatches was allowed up to 2. High-quality sequences were de-noised using the DADA2^55^ plugin in the QIIME2 (version 2020.2)^56^ pipeline with default parameters to obtain amplicon sequence variants (ASVs). The taxonomy of the ASVs was assigned using the SILVA reference database. Reads classified as mitochondria or chloroplasts were removed before the analysis. To minimize the effects of sequencing depth on alpha and beta diversity measures, the original ASV table was rarefied according to the sample with the lowest number of reads (2861).

Microbial data were analyzed using the Majorbio Cloud Platform (www.majorbio.com). In brief, alpha diversity indices were calculated utilizing the Mothur software (version 1.30.2). PCoA was performed using the R package vegan (version 3.3.1), based on abundance-weighted Jaccard distances. To determine the statistical significance of differences in taxonomic and functional profiles between two groups or among multiple groups, the Mann – Whitney U test or the Kruskal-Wallis test was used, along with FDR p-value correction, using the R package stats. Additionally, the Linear Discriminant Analysis (LDA) Effect Size (LEfSe) method was used to identify significant differences in relative abundance between groups, with an LDA score threshold of 2. Correlations between genus-level abundance and both clinical and immune markers were explored by Spearman analysis using the R package pheatmap. The microbial community functions were predicted using FAPROTAX (version 1.2.1) and BugBase (https://bugbase.cs.umn.edu/index.html).

### mRNA sequencing

RNA extraction, sequencing, and analysis were performed by Majorbio Co. Ltd. (Shanghai, China). All samples were analyzed in the same batch to avoid between-batch variability. Total RNA was isolated using TRIzol® reagent according to the manufacturer’s instructions. The RNA-seq library was constructed using the Illumina TruSeq® stranded mRNA kit (Illumina). Library fragments were sized and selected to obtain fragments of 300 bp using 2% agarose gel electrophoresis. The selected fragments were amplified by PCR for 15 cycles and sequenced using a NovaSeq 6000 sequencer (Illumina). RNA sequencing data were deposited in the Sequence Read Archive database under PRJNA number 1,035,440.

Raw paired-end reads were trimmed and quality controlled using fastp^54^ with default parameters. Subsequently, the clean reads were individually aligned to the reference genome in orientation mode using HISAT2^57^ software. The cleaned reads had a mean error rate of ≤ 0.03% and a Q30 score of ≥ 93% for all samples. More than 95% of the reads were mapped and > 89% of the mapped reads were unique. The mapped reads for each sample were assembled using StringTie^58^ with a reference-based approach. To identify differentially expressed genes (DEGs) between two different samples, the expression level of each transcript was calculated based on the transcripts per million reads (TPM) method. RSEM^59^ was used to quantify the gene abundance. All RNA sequencing data were analyzed using the Majorbio Cloud Platform (www.majorbio.com).

### Histology

Liver tissues from mice were immediately fixed in 4% paraformaldehyde, embedded in paraffin, sliced into 2-μm sections, and stained with hematoxylin and eosin (H&E). Histological features of MAFLD, including steatosis, lobular inflammation, and ballooning, were scored according to the NAFLD activity scoring system.^60^

### Immunofluorescence

Deparaffinized sections of the human liver were subjected to heat-induced antigen retrieval. Subsequently, the sections were treated with PBS containing 5% BSA and 0.2% Triton X-100 at room temperature for 30 min. After blocking, sections were incubated overnight at 4°C with primary antibodies against RORγt (1:100; eBioscience, 14-6988-82) and CD3 (1:100; Abcam, ab5690). Following extensive washing with PBS, the sections were incubated with CY3- or Alexa Fluor 488-labeled secondary antibodies. Nuclei were counterstained with DAPI, and images were captured using a Pannoramic MIDI fluorescence microscope.

### Enzyme-Linked Immunosorbent Assay (ELISA)

ELISA was conducted using mouse IL-2 (CSM-0701 M2; New Cell & Molecular Biotech), IL-17 (JM-02453 M2; Jingmei Biotech), IL-22 (JM-11424 M2; Jingmei Biotech), lymphotoxin β (CSM-0821 M2; New Cell & Molecular Biotech), and GM-CSF (CSM-0185 M2; New Cell & Molecular Biotech) ELISA kits following established protocols.

### Biochemical indicator analyses

Plasma samples were collected by centrifuging heparinized blood at 2000 × g for 20 min. Liver tissues were homogenized in PBS at 0.1 g tissues/mL of PBS. The levels of ALT, AST, triglyceride, and total cholesterol were detected using a Chemistry Analyzer (Beckman AU5800).

### Statistical analyses

Statistical analyses were performed using Prism 7.0 (GraphPad Prism). Continuous variables were expressed as mean ± standard deviation or median with interquartile ranges. Categorical variables were expressed as numbers and percentages. Comparisons between two groups were performed using Student’s t-tests (paired or unpaired, as appropriate), Mann-Whitney rank sum tests, or Chi-square tests. Correlations were evaluated using Pearson’s or Spearman’s tests. A two-tailed P-value threshold of < .05 was considered statistically significant.

## Supplementary Material

240325 Supplementary material clean.docx

## Data Availability

The data that support the findings of this study are available in https://www.ncbi.nlm.nih.gov/bioproject/PRJNA1035094., reference number PRJNA1035094, https://www.ncbi.nlm.nih.gov/bioproject/PRJNA1035440, reference number PRJNA1035440, and within the article and its supplementary materials.
