# Supplementary material for: Washed microbiota transplantation promotes homing of group 3 innate lymphoid cells to the liver via the CXCL16/CXCR6 axis: a potential treatment for metabolic-associated fatty liver disease
Source: Gut Microbes. 2024 Jun 28;16(1):2372881. doi: 10.1080/19490976.2024.2372881 (PMC11216104; doi:10.1080/19490976.2024.2372881)
Supplement: 240325 Supplementary material clean.docx [file KGMI_A_2372881_SM8936.docx]

**Supplementary material**


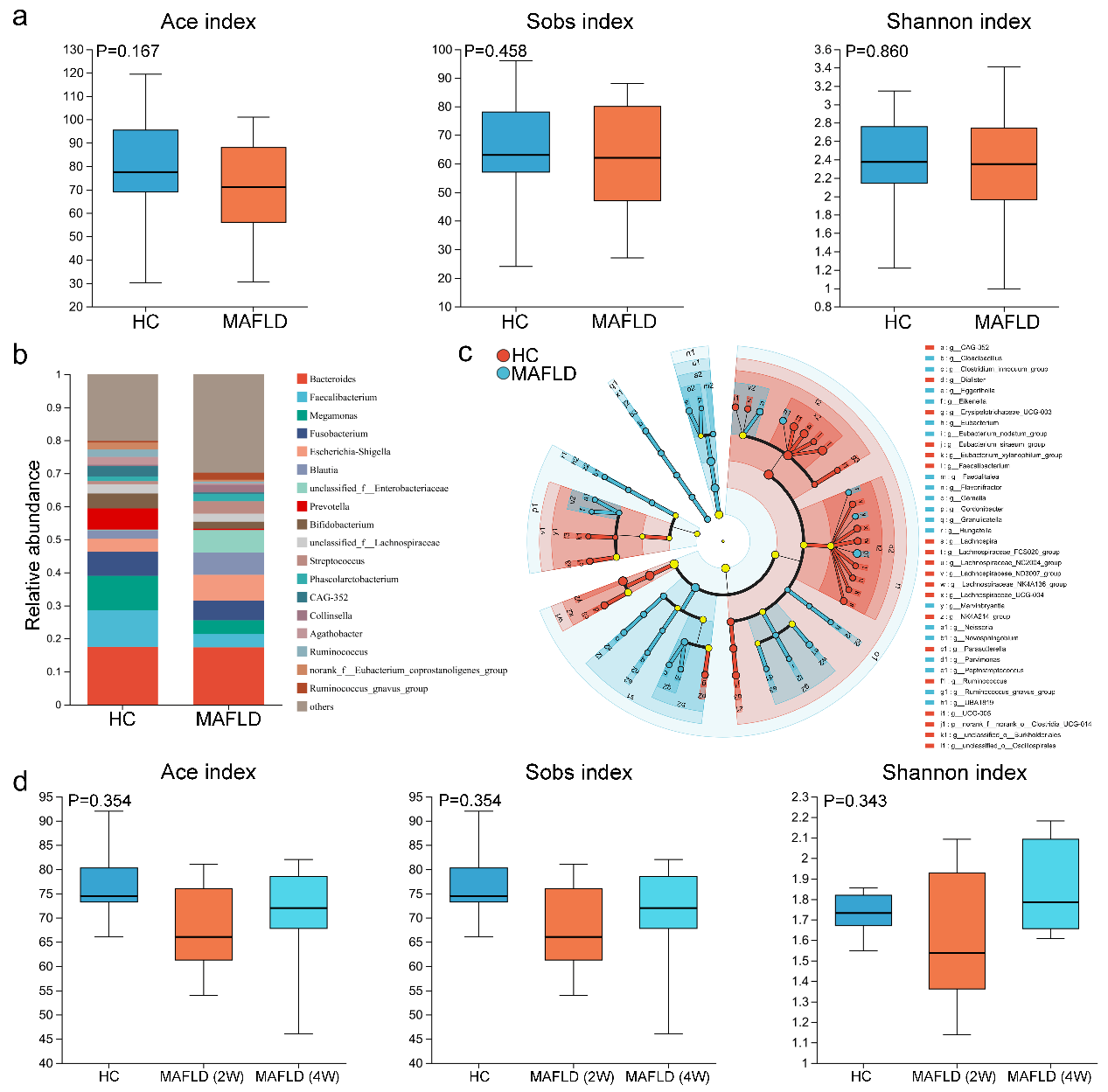


**Figure S1.** The composition of the gut microbiota was altered in both MAFLD patients and mice. (a) Box plots comparing gut microbiota alpha diversity indices (Ace, Sobs, and Shannon) in healthy controls and patients with MAFLD. (b) Bar charts comparing gut microbial communities at the genus level between healthy controls and patients with MAFLD. (c) Linear discriminant analysis effect size (LEfSe) comparison of the gut microbiota between healthy controls and MAFLD patients (p < 0.05, LDA > 3.5). (d) Box plots showing alpha diversity indices (Ace, Sobs, and Shannon) of the gut microbiota in healthy (HC), MAFLD-2W, and MAFLD-4W mice. MAFLD, metabolic-associated fatty liver disease.

**
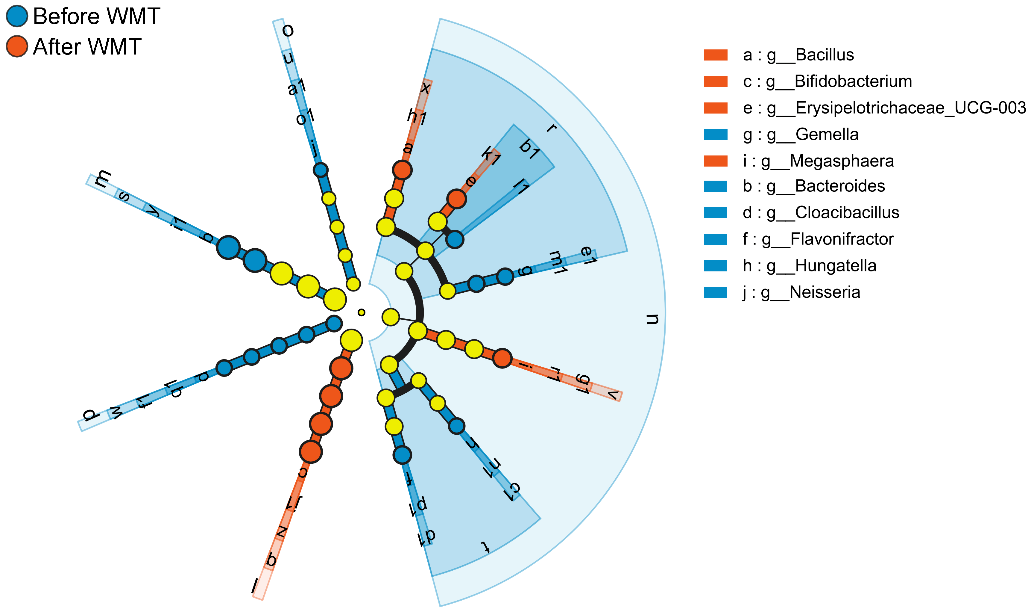
**

**Figure S2.** LEfSe comparison of the gut microbiota in MAFLD patients before and after WMT.


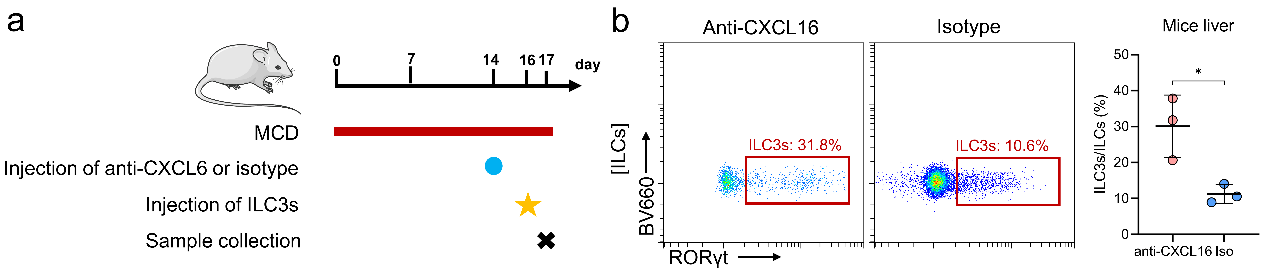


**Figure S3.** Blocking CXCL16 inhibited the migration of ILC3s to the liver in MAFLD mice. (a) Study design for CXCL16 blocking experiments. (b) Proportion of ILC3s in the livers of MAFLD mice treated with CXCL16-neutralizing antibody or isotype IgG control after receiving ILC3s (n = 3/group). ILC3, type 3 innate lymphoid cells; MAFLD, metabolic-associated fatty liver disease; MCD, methionine- and choline-deficient diet. *p < 0.05.


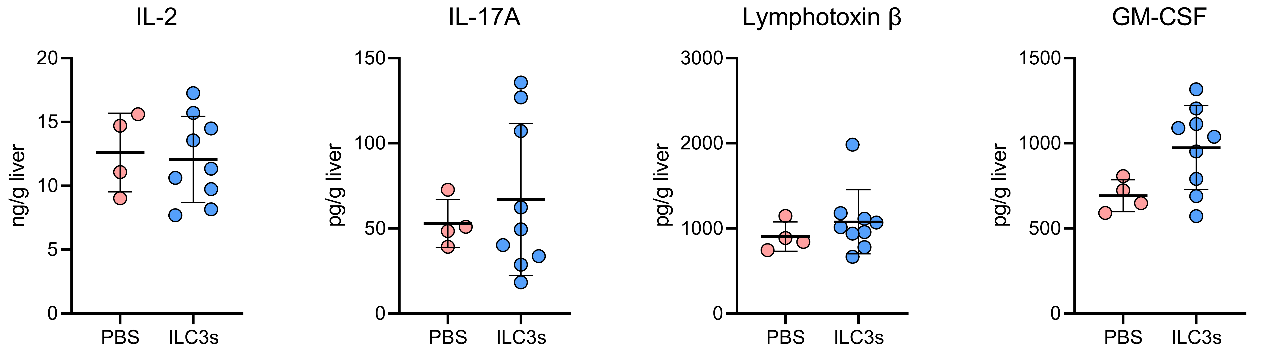


**Figure S4.** Concentrations of other ILC3-related cytokines in the livers of MAFLD mice treated with ILC3s or PBS.

**Table S1.** Characteristics of patients with MAFLD who received WMT or conventional therapy.

|  | WMT group  (N = 59) | Conventional therapy group  (N = 84) | P-value |
| --- | --- | --- | --- |
| Male sex (%) | 34 (57.6) | 48 (57.1) | 1.000 |
| Age (years) | 58.0 (41.0–64.0) | 59.5 (50.0-67.0) | 0.015 |
| Fat attenuation parameter (dB/m) | 272.3 ± 31.1  (n = 38) | 272.6 ± 26.6  (n = 30) | 0.971 |
| Serum cholesterol (mmol/L) | 4.9 (4.2–5.7)  (n = 52) | 5.1 (4.2–5.6)  (n = 79) | 0.817 |
| Serum triglyceride (mmol/L) | 1.7 (1.1–2.5)  (n = 52) | 2.1 (1.1-3.0)  (n = 79) | 0.169 |

Data are presented as the mean ± standard deviation, median (interquartile range), or n (%). WMT, washed microbiota transplantation.
